# Ocular surface glycocalyx in health and disease

**DOI:** 10.3389/fcell.2025.1561324

**Published:** 2025-03-27

**Authors:** Rafael Martínez-Carrasco, Ajay Sharma

**Affiliations:** ^1^ Department of Ophthalmology, Tufts Medical Center, Tufts University School of Medicine, Boston, MA, United States; ^2^ Chapman University School of Pharmacy, Chapman University, Irvine, CA, United States

**Keywords:** glycocalyx, ocular surface, glycosylation, membrane-associated mucin, dry eye disease, infection, GVHD

## Abstract

The glycocalyx is a dynamic carbohydrate-enriched structure that forms a dense coating on the surface of animal cells, including those at the ocular surface. It plays a critical role in maintaining cellular functions and it has a significant influence in disease processes. At the ocular surface, glycoproteins such as mucins are essential for hydration, lubrication, and barrier protection. Proteoglycans and glycolipids contribute to cell signaling, and mediate interactions with pathogens. Alterations in the glycocalyx are implicated in a spectrum of ocular conditions, including dry eye disease, ocular allergies, infections, and systemic diseases such as Graft-versus-host disease (GVHD) and diabetes mellitus. Research has revealed alterations in mucin expression and aberrant glycosylation but many aspects of how these alterations contribute to disease processes remain poorly understood. Advancing our knowledge of glycocalyx composition and function offers valuable insights into the pathophysiology of ocular surface diseases and presents opportunities for novel glycocalyx-targeted therapeutic strategies to mitigate disease progression and enhance patient care. This review explores the current knowledge on the composition and functions of the ocular surface glycocalyx, emphasizing its implications in ocular surface disease.

## 1 Introduction

The glycocalyx is a dynamic carbohydrate-enriched structure that forms a dense coat on the external surface of all animal cells (Varki and Kornfeld, 2022). The term “glycocalyx” is derived from the Greek words “glykos,” signifying “sweet,” and “kalyx,” which translates to “husk”. The concept was first introduced by Bennett in 1963 as a descriptor for the sugar-containing structures observed on the surfaces of various cellular bodies (Bennett, 1963). This pericellular layer, undergoes compositional changes during cell growth, differentiation, and disease progression, making the glycocalyx a constant yet continuously changing feature of the cellular landscape (Stanley, 2024). The glycocalyx consists of polysaccharide structures covalently linked to proteins, lipids, or RNA, to form glycoproteins, proteoglycans, glycolipids, and glyco-RNAs (Flynn et al., 2021). The addition of sugar moieties through glycosylation is essential for the proper function of these biomolecules, affecting their stability, enzymatic activity, and precise localization at the cell membrane.

The dynamic nature of the glycocalyx allows it to rapidly respond to stress and disease processes, making it a valuable source of disease biomarkers (Rudman et al., 2019; Verhelst et al., 2020). Alterations in the glycocalyx in response to stress frequently lead to disruptions in cellular function, which can be the direct cause of disease. Consequently, the glycocalyx has emerged as a promising target for therapeutic interventions to mitigate disease progression (Rodriguez et al., 2018). In this review, we will explore the current understanding of the ocular glycocalyx, its various roles in cellular functions, and its potential implications in health and disease.

## 2 The ocular surface glycocalyx

Coating the surface of the eye are the specialized epithelia of the cornea and conjunctiva, overlaid by a tear film that hydrates the eye and maintains a smooth, refractive surface. Exposed to a dry and hostile environment, this surface relies on the glycocalyx to provide a protective barrier and retain the tear film’s moisture. Alterations in the glycocalyx are evident in ocular surface diseases, with reduced mucin expression and glycosylation changes contributing to epithelial barrier disruption and inflammation. These changes exacerbate disease progression by promoting immune activation, tear film instability, or pathogen adhesion.

The abundant glycoconjugates that populate the cell surface serve as a protective barrier against pathogens, preventing their direct contact with the cell membrane and inhibiting their ability to invade the cell (Argueso et al., 2021). However, the functionality of the glycocalyx extends beyond its role as a protective shield (Varki, 2017). The glycocalyx actively participates in several cellular functions and plays a crucial role in mediating cell-cell communication. As cellular receptors, the glycocalyx facilitates intracellular signaling pathways, thereby influencing several aspects of cell behavior such as growth, differentiation, and response to external stimuli.

**FIGURE 1:**
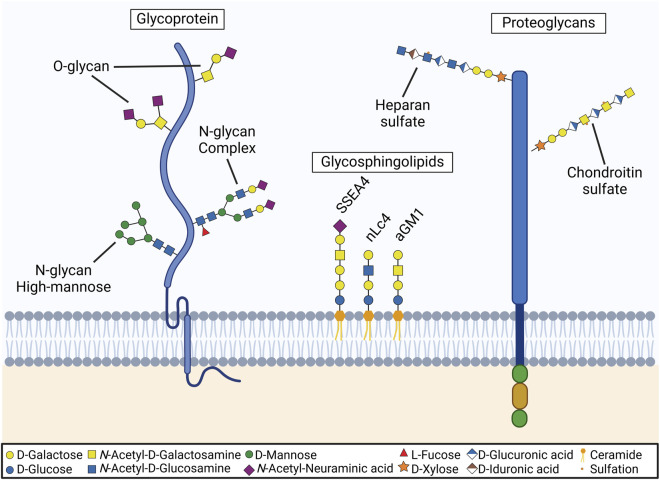
Components of the ocular surface glycocalyx. Diagram illustrating the main glycans present on the ocular surface. Glycoproteins primarily feature core 1 sialylated O-glycans, high-mannose N-glycans, and complex N-glycans. Glycosphingolipids include Stage-Specific Embryonic Antigen-4 (SSEA4), neolactotetraosylceramide (nLcn4), and asialotetrahexosylganglioside (aGM1). Proteoglycans are mainly modified with chondroitin sulfate and heparan sulfate.

The synthesis of the glycocalyx occurs through glycosylation, a fundamental cellular process wherein carbohydrate chains (glycans) are covalently attached to proteins and lipids. This highly intricate enzymatic process begins in the endoplasmic reticulum, where nascent glycan structures are assembled, and is completed in the Golgi apparatus, where these structures undergo modifications to form mature and functionally diverse glycans. For a deeper understanding of this process, several comprehensive reviews are available (Schjoldager et al., 2020; Moremen et al., 2012). Like all cells, ocular surface cells utilize a diverse array of glycosylation pathways, each contributing to the synthesis of distinct glycoconjugates in a tightly regulated manner. The activation of these pathways and the specific glycosyltransferase enzymes involved orchestrate the composition and structural diversity of the glycocalyx. Elucidating which glycosylation pathways are active and identifying the enzymes present are crucial steps in uncovering the glycocalyx’s functions and its roles in ocular surface health and disease. Figure 1 illustrates the major structural components of the ocular surface glycocalyx, which include glycoproteins, containing N- and O-glycans, proteoglycans and glycosphingolipids. In the following sections we will delve into the knowledge of the specific structures and pathways found in the ocular surface.

**TABLE 1:** Changes in glycocalyx in ocular surface disease. Upward and downward arrows represent upregulation and downregulation, respectively. A dash signifies no observed change. WGA-L, PNA-L, and SBA-L refer to changes in the ligands specific to these lectins.

Disease	Glycocalyx-related changes	Reference
Sjögren DED	↓MUC1	Jones et al. (1998)
	– MUC1 – MUC4	Argueso et al. (2002)
	↑MUC1 ↑MUC16	Caffery et al. (2008) Caffery et al. (2010) Srinivasan et al. (2013)
Non-Sjögren DED	↓MUC1 ↓MUC4	Corrales et al. (2011)
	– MUC1 – MUC4	Caffery et al. (2008) Caffery et al. (2010)
	↑MUC1 ↑MUC4 ↑MUC16	Choi and Tichenor (2024) Gipson et al. (2011)
	↓WGA-L ↓PNA-L ↓SBA-L	Versura et al. (1986)
	↑Free Gal-3	Uchino et al. (2015)
Contact lens wear	– MUC1 – MUC4 – MUC16	Hori et al. (2006)
	↓MUC4 ↓MUC16	Corrales et al. (2009)
	↓WGA-L	Fukui et al. (2016) Read et al. (2020)
Multipurpose solution	↓MUC1 ↓MUC4 ↓MUC16	Imayasu et al. (2010) Tchedre et al. (2013) Gordon et al. (2011)
Allergy	↑MUC1 ↑MUC4	Dogru et al. (2006)
	↑MUC1 ↑MUC4	Hu et al. (2007)
	↑MUC16	Dogru et al. (2006)
	↑Muc4	Kunert et al. (2001)
	↓High-mannose N-glycan ↓Bisecting N-glycan ↑Sialyl bi- and multi-antennary N-glycan	Messina et al. (2021)
	↑Free Gal-3	Ito et al. (2023)
GVHD	↓Muc4	Shamloo et al. (2019)
MMP	↓GALNTs	Argueso et al. (2003)
	↓Bi- and multi-antennary N-glycan ↓MGAT1 ↓MGAT2 ↓MGAT4B	Woodward et al. (2019)
Diabetes	↓WGA-L	Weng et al. (2022)

### 2.1 Glycoproteins at the ocular surface glycocalyx

Formed by the conjugation of carbohydrate groups to polypeptide chains, glycoproteins are integral components of the glycocalyx on the ocular surface. Undoubtedly, the most widely studied components of the ocular surface glycocalyx are membrane-associated mucins (MAMs). The mucin family is distinguished by the presence of structural module(s) containing numerous tandem repeats rich in serine and threonine residues that serve as sites for O-linked oligosaccharide chains. With glycans accounting for 50%–90% of the mass of the molecule, these glycoproteins are by far the largest component of the ocular glycocalyx, reaching more than 2 MDa (Perez and Gipson, 2008). To date, 6 MAMs have been found forming part of the human ocular surface glycocalyx: MUC1, MUC4, MUC16, MUC20, MUC21 and MUC22 (Fini et al., 2020). The densely packed glycan chains confer the biophysical properties of hydration, lubrication, anti-adhesion, and repulsion needed for the diverse functions of mucins, which include barrier function, regulation of inflammation, and desquamation.

Each MAM is primarily located at the apical aspect of the ocular surface, anchored to membrane expansions known as microplicae. The distribution of these molecules varies significantly between the corneal and conjunctival epithelia, with some MAMs being more prevalent in the cornea and others in the conjunctiva. Furthermore, this distribution pattern differs across species. In humans, MUC1 and MUC16 are present across both the corneal and conjunctival surfaces. A recent study found that MUC1 expression is significantly higher in the nasal conjunctiva compared to the superior and inferior bulbar regions (Choi and Tichenor, 2024). MUC4 is found in the conjunctiva and peripheral cornea, decreasing towards the central cornea (Inatomi et al., 1996). In contrast, in mice, there is very little Muc1 in the corneal epithelium but high levels present in the conjunctival epithelium (Kardon et al., 1999). Muc4 is present throughout the murine corneal and conjunctival epithelia, showing a linear pattern along apical cells (Lange et al., 2003; Danjo et al., 2000). This pattern is similar in rat ocular surface epithelia (Swan et al., 2002; Price-Schiavi et al., 1998). Moreover, Muc16 in mice is only found at the apical aspect of the conjunctival epithelium and on goblet cells (Wang et al., 2008; Shirai et al., 2014), with no presence in the corneal epithelium. As a result, the corneal epithelial surface MAM length in mice is about four times shorter than in humans. MUC16 plays the main barrier role in the human cornea, while Muc4 appears to fulfill this function in mice (Martinez-Carrasco et al., 2023). MUC21 and MUC22 proteins have been identified in the human corneal epithelium, but their presence in the conjunctiva has not yet been examined, nor has their ocular distribution in other species been studied (Fini et al., 2020).

Beyond mucins, many other proteins at the cell surface of epithelial cells are glycosylated. Most of the proteins within the secretory pathway undergo glycosylation, suggesting that the majority of cell surface receptors are subject to this modification (Steentoft et al., 2013; Zielinska et al., 2010). This modification is pivotal for receptor functionality. Key glycoproteins involved in proliferation, differentiation, and migration include the Epidermal Growth Factor Receptor (EGFR), Notch receptor, CD147, and α3β1 integrin. EGFR has an extensively N-glycosylated extracellular domain, which regulates growth factor binding, conformation, and function (Zhen et al., 2003; Zielinska et al., 2010; Fernandes et al., 2001; Kaszuba et al., 2015). Glycosylation, particularly O-glycosylation, is also crucial for regulating Notch signaling (Stanley, 2007). Both CD147 and α3β1 integrin contain N-glycan modifications that bind to galectin-3 and are essential for their roles in wound healing (Saravanan et al., 2009; Mauris et al., 2014; Gonzalez-Andrades et al., 2020).

#### 2.1.1 Protein glycosylation in the ocular surface

The diverse forms of protein glycosylation are primarily characterized by the bond between the sugar and the protein, as well as the initial monosaccharide linked to the proteins. Glycans are attached to proteins in several ways: N-linked to asparagine (Asn), O-linked to the hydroxyl groups of serine (Ser), threonine (Thr), or tyrosine (Tyr), C-linked to tryptophan (Trp), and through glypiation. Various O-linked sugars include N-Acetylgalactosamine (GalNAc), Fucose (Fuc), N-Acetylglucosamine (GlcNAc), Mannose (Man), Glucose (Glc), Xylose (Xyl), and Galactose (Gal) (Schjoldager et al., 2020). Pathways include 14 distinct types of protein glycosylation, including N-glycosylation and 11 types of O-glycosylation. The initial attachment of glycans to proteins and their final composition is largely dependent on the repertoire of glycogenes in each cell. Hence, glycosylation signatures can vary significantly between different tissues and cell types. In the ocular surface, this implies a difference in the glycans that are found in the various compartments (conjunctiva, cornea, or tear film), the functional consequence of which has not yet been studied. Furthermore, these glycan structures can undergo alterations in states of disease that regulate the levels of glycogenes, as will be discussed in the sections below.

##### 2.1.1.1 N-glycans at the ocular surface

**FIGURE 2:**
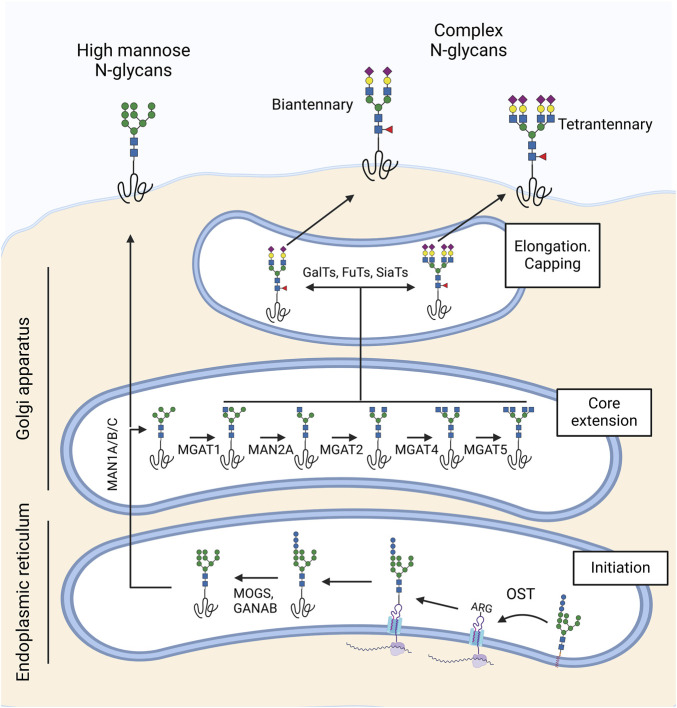
Synthesis of N-glycans in the ocular surface. N-glycosylation begins in the endoplasmic reticulum with the oligosaccharyltransferase (OST) complex, which attaches an oligosaccharide precursor from dolichol phosphate to asparagine (Asn) in the Asn-X-Ser/Thr consensus sequence. MOGS and GANAB glycosidases then remove three terminal glucose residues, forming a high-mannose N-glycan structure. In the Golgi apparatus, α-mannosidases (MAN1A/B/C, MAN2A) and β-GlcNAc transferases (MGAT1, MGAT2, MGAT4, MGAT5) synthesize hybrid and complex structures with varying numbers of antennae. Finally, galactosyl-transferases, fucosyl-transferases, and sialyl-transferases extend and cap the N-glycan. The ocular surface predominantly features biantennary and multiantennary N-glycans rich in sialic acid and core fucose.

N-glycosylation involves the attachment of a dolichol phosphate-linked oligosaccharide precursor to the amide group of asparagine (Asn) in the consensus peptide sequence Asn-X-Ser/Thr, where X can be any amino acid except proline. This process is initiated in the endoplasmic reticulum by the enzymatic complex oligosaccharyltransferase (OST) (Figure 2). Following this, glycosidases remove three terminal glucose residues, leaving a glycan structure terminated in nine mannose residues, which forms the basis for the high-mannose type of N-glycan structures. The further action of α-mannosidases in the Golgi apparatus and subsequent addition of β-GlcNAc initiates the synthesis of hybrid and complex structures with different numbers of antennae. Finally, the addition of galactose, fucose and sialic acid by Golgi transferases further modifies these glycans before the glycoprotein is transported to the plasma membrane.

The ocular surface exhibits a diverse array of N-glycans, including high-mannose and complex structures, with some regional differences in their distribution. Lectin-based studies have demonstrated the presence of high-mannose glycans in the corneal epithelium, as shown by staining with mannose-specific lectins such as Concanavalin A (ConA) and *Galanthus nivalis* agglutinin (GNA) (Brandon et al., 1988; Bishop et al., 1991; Mencucci et al., 2011). A lectin array analysis of human corneal cell surface proteins further revealed an enrichment of high-mannose glycans in the central cornea compared to the limbal epithelium (Martinez-Carrasco and Argueso, 2023). Additional lectin staining with *Datura stramonium* agglutinin (DSA), *Phaseolus vulgaris* leukoagglutinin (PHA-L), and *P. vulgaris* erythroagglutinin (PHA-E) has identified complex N-glycans in the corneal epithelium (Mencucci et al., 2011; Argueso et al., 1998; Brandon et al., 1988; Bishop et al., 1991).

Mass spectrometry studies have added further detail to this glycan landscape, characterizing N-glycans in tear fluid and transmembrane mucin-enriched samples of a corneal epithelial cell line. Tear fluid contains over 50 major N-glycans, predominantly complex types, with a notable prevalence of structures containing bisecting GlcNAc residues (Nguyen-Khuong et al., 2015; Rodriguez Benavente and Argueso, 2018). In contrast, N-glycans on transmembrane mucins of differentiated human corneal epithelial cells are primarily complex-type, biantennary or multiantennary, enriched with N-acetyllactosamine, a key ligand for galectins, and capped with sialic acid (Taniguchi et al., 2017). In addition, qPCR analysis has confirmed that both corneal and conjunctival epithelial cells express glycosyltransferases involved in the biosynthesis of N-glycan branching structures in the medial Golgi. These include N-acetylglucosaminyltransferases I, II, IV, and V (MGAT1, MGAT2, MGAT4, and MGAT5), which are required for generating hybrid and complex N-glycans, as well as α-mannosidase II enzymes (MAN2A) that modify high-mannose N-glycan precursors. Some of these enzymes showed differences in expression levels between conjunctival and corneal epithelial cells. Nevertheless, how these variations translate into differences in N-glycan structures and their functional implications for each compartment remains unexplored.

The N-glycans of the ocular surface glycocalyx play crucial roles in maintaining barrier function and regulating cell migration during re-epithelialization. These functions are closely related to the interaction of lactosamine-containing N-glycans with the galactose-binding lectin galectin-3. The elimination of N-glycans using PNGase F and the silencing of *MGAT1*, which disrupts the first step in antennary formation, reduce the binding of galectin-3 to MUC16 and increase Rose Bengal penetrance *in vitro* (Taniguchi et al., 2017). Similarly, the abrogation of N-glycan extension decreases the interaction between CD147 and galectin-3, affecting the ability of CD147 to cluster and promote epithelial remodeling and migration (Gonzalez-Andrades et al., 2020). Additionally, galectin-3 binding to the N-glycans present on α3β1 integrin initiates a signaling cascade that directs lamellipodia formation, which is crucial for epithelial migration (Saravanan et al., 2009).

##### 2.1.1.2 O-glycans at the ocular surface

**FIGURE 3:**
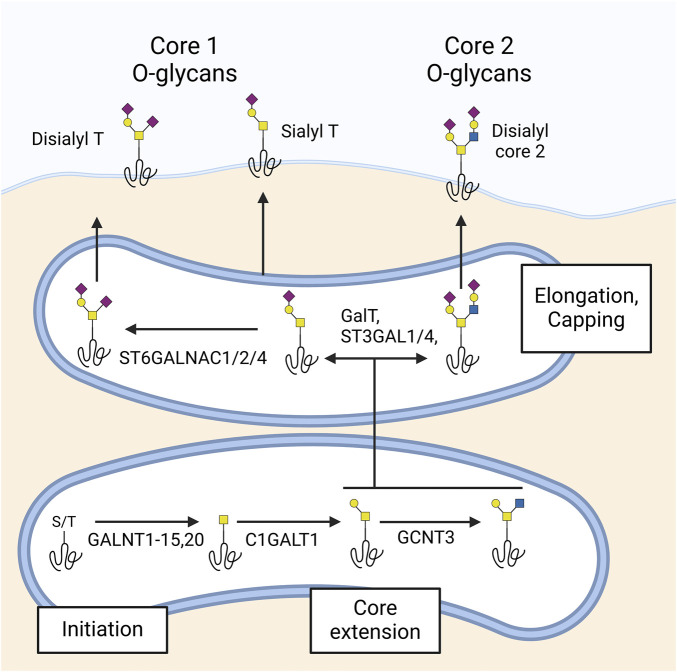
Synthesis of O-glycans in the ocular surface. O-glycosylation begins in the Golgi with polypeptide GalNAc transferases (GALNTs) acting on serine (Ser) or threonine (Thr) residues, producing the Tn antigen. At the ocular surface, GALNTs 1–15 and 20 have been detected. The Tn antigen is extended by β1,3-galactosyltransferase (C1GALT1) to form the T antigen, which can be further extended to core 2 by β1,6 N-acetylglucosaminyltransferase (GCNT3). Additionally, β-galactoside α2-3-sialyltransferases 1 and 4 (ST3GAL1 and 4) and α-N-acetylgalactosaminide α2-6-sialyltransferases 1, 2, and 4 (ST6GALNAC1, 2, and 4) are responsible for capping core 1 and core 2 glycans with sialic acid. The ocular surface predominantly features sialyl- and disialyl core 1 (sialyl and disialyl T antigen) and disialyl core 2 glycans.

O-glycosylation entails the attachment of a single carbohydrate to the hydroxyl groups of serine (Ser) or threonine (Thr) residues. Among several types of O-glycosylation, O-GalNAc glycosylation, also known as mucin-type O-glycosylation, is the most common and diverse. This process is initiated in the Golgi apparatus by up to 20 polypeptide GalNAc transferase (GALNT) isoenzymes with partially overlapping specificities (Schjoldager et al., 2020). The initial O-GalNAc structure can be further modified by the addition of other sugars, leading to the formation of 8 known core structures. At the ocular surface, only core 1 and core 2 O-glycans have been detected (Figure 3). Similar to N-glycans, O-glycans are further extended and capped by the addition of galactose, fucose, and sialic acid.

O-GalNAc glycans are abundantly present on the ocular surface epithelium, with distinct differences in composition and biosynthesis observed across regions and cell types. Lectin array analyses of corneal epithelial cell surface proteins reveal strong binding of O-glycan-specific lectins such as BPA and DISCOIDIN-I, indicating a prominent presence of these glycans (Martinez-Carrasco and Argueso, 2023). Histological studies further confirm that the epithelial glycocalyx of the cornea contains sialylated core 1 O-glycans. In neuraminidase-treated paraffin sections of human, cat, and rabbit corneas, the lectin PNA stains the entire epithelium, implying the presence of sialylated core 1 O-glycans on the epithelial glycocalyx (Panjwani et al., 1986).

Mass spectrometry analyses revealed that O-GalNAc glycans at the ocular surface are relatively small compared to those in other mucosal secretions, yet they exhibit considerable structural diversity. In the human conjunctiva, over 70% of the O-GalNAc glycans are core 1-based structures, including core 1, α2-3 sialyl core 1, and disialyl core 1 (Rodriguez Benavente and Argueso, 2018). Notable differences in glycan composition exist between the conjunctiva and the tear film. For instance, disialyl core 1 and core 2-based structures are present in the conjunctival epithelium but absent in the tear film (Royle et al., 2008). Additionally, α2-3 sialyl core 1 dominates in conjunctival tissue, whereas α2-6 sialyl core 1 is the primary glycan in tears (Guzman-Aranguez and Argueso, 2010). These differences might suggest unique roles for these mucin-type O-glycans in different compartments of the ocular surface.

The diversity of O-GalNAc glycans at the ocular surface is shaped by the expression patterns of glycogenes, particularly the GALNT family of enzymes responsible for initiating O-GalNAc glycosylation. Glycogene microarrays and real-time PCR studies have shown that the human conjunctival epithelium expresses 16 different GALNTs, including GALNT1–15 and GALNT20 (Guzman-Aranguez et al., 2009; Guzman-Aranguez and Argueso, 2010; Imbert et al., 2006). Immunohistochemical studies reveal that these enzymes are distributed in a cell layer- and cell type-specific manner, with GALNT4 localized to apical cell layers, GALNT2 to basal cell layers, and GALNT6 exclusively to conjunctival goblet cells. This enzyme specificity is critical, as the repertoire of GALNTs expressed by a given cell determines its O-glycoproteome. Supporting evidence from studies in other epithelial systems, such as skin keratinocytes, shows that distinct GALNTs target unique substrates, influencing processes such as cell-matrix adhesion and epithelial differentiation (Bagdonaite et al., 2020).

Glycogene microarrays in human conjunctiva have also demonstrated the expression of β1,3-galactosyltransferase (T-synthase; C1GALT1), responsible for the biosynthesis of core 1, and β1,6 N-acetylglucosaminyltransferase (GCNT3), which synthesizes core 2, aligning with mass spectrometry findings. Additionally, β-galactoside α2-3-sialyltransferases 1 and 4 (ST3GAL1 and 4) and α-N-Acetylgalactosaminide α2-6-sialyltransferases 1, 2 and 4 (ST6GALNAC1, 2 and 4) were also found in the conjunctiva; these enzymes are responsible for the capping of core 1 and core 2 glycans with sialic acid (Guzman-Aranguez et al., 2009). Abrogation of O-glycan core extension by silencing C1GALT1 causes a loss of epithelial barrier function *in vitro*, underscoring the importance of O-glycans in maintaining this function (Argueso et al., 2009).

### 2.2 Proteoglycans

Proteoglycans are another major component of the glycocalyx. These macromolecules consist of a protein core with one or more glycosaminoglycan (GAG) chains covalently bonded. GAGs are extensive linear polysaccharides consisting of repeating disaccharide units, each containing an amino sugar paired with either a uronic acid or galactose. These GAG chains vary in structure and attachment, contributing to the diversity of proteoglycans. Proteoglycans are ubiquitously produced by virtually all mammalian cells and can be secreted into the extracellular matrix (ECM), inserted into the plasma membrane, or stored in secretory granules. Contrary to the extensive variety of glycoproteins bearing N- and O-linked glycans, only a relatively small number of proteins, fewer than 50, have been identified to carry GAG chains (Merry et al., 2022).

Keratan sulfate (KS) is a type of GAG chain, with two distinct forms: KS I and KS II. KS I, initially identified in the cornea, is linked to protein through an asparagine residue via an N-glycan. In contrast, KS II, also known as skeletal keratan sulfate, is attached through an O-glycan core 2 structure via N-acetylgalactosamine to serine or threonine. Two other significant classes of GAG chains are chondroitin sulfate/dermatan sulfate and heparan sulfate/heparin, which are attached to serine residues in proteins through a xylose linkage. The initiation of this linkage is catalyzed by xylosyltransferase, which uses UDP-xylose as a donor. In vertebrates, two isoforms of xylosyltransferase, XYLT1 and XYLT2, are known (Schjoldager et al., 2020). Following xylose addition, a linkage tetrasaccharide is formed by the sequential action of specific β4 galactosyl-, β3 galactosyl-, and β3 glucuronosyltransferase enzymes. This tetrasaccharide acts as a branching point for GAG biosynthesis, leading to the addition of either β4-linked N-acetylgalactosamine, initiating chondroitin sulfate assembly, or α4-linked N-acetylglucosamine, starting heparan sulfate assembly. Hyaluronan, another GAG, differs from others as it does not covalently attach to a core protein but instead interacts non-covalently with proteoglycans through hyaluronan-binding motifs. A study utilizing immunogold labeling to localize keratan sulfate, chondroitin sulfate, and heparan sulfate in human corneas revealed that heparan sulfate and chondroitin sulfate are the predominant glycosaminoglycans in the epithelium (Figure 1), whereas keratan sulfate is mainly found in the stromal extracellular matrix and basal membranes (Bairaktaris et al., 1998). For a more comprehensive understanding, readers can refer to Puri et al., 's 2020 review on the distribution of proteoglycans in the cornea (Puri et al., 2020).

Syndecans (SDCs) consist of four members, each featuring a short hydrophobic domain that spans the membrane, connecting the extracellular domain, which has GAG attachment sites, to a smaller intracellular cytoplasmic domain. Transcripts for each of the four syndecan isoforms are found in human corneal epithelial cell culture, with SDC4 and SDC1 being the most abundant, and SDC2 the scarcest (Garcia et al., 2016). SDC1 plays a multifaceted role in corneal health and disease. It regulates epithelial cell proliferation, integrin expression, migration, and wound healing. Deficiency in SDC1 in mice leads to defects in these processes, particularly affecting corneal epithelial cell migration and proliferation rate after wounding (Stepp et al., 2002). Interestingly, lacritin, a protein that promotes homeostasis in corneal epithelial cells, targets SDC1 on cell surfaces, aiding in cellular protection and metabolic balance (Wang et al., 2013).

Glypicans, another family of cell-surface proteoglycans, are characterized by carrying only heparan sulfate (HS) chains. The glypican family in mammals comprises six members, each anchored to the outer leaflet of the plasma membrane through a glycosylphosphatidylinositol (GPI) anchor at the carboxyl terminus. Not all isoforms of glypicans were detected in human corneal epithelial cells in culture, with GPC1 being the most abundant species (Garcia et al., 2016). Glypican-4 is found in endothelium and fibroblasts (Cheong et al., 2013), while glypican-3 is present in mouse corneal epithelium and increases during wound healing (Saravanan et al., 2010).

CSPG4, also known as Chondroitin Sulfate Proteoglycan 4, is a type of proteoglycan that is expressed by non-myelinating Schwann cells located in the corneal stroma. A recent study suggested that CSPG4 is associated with a faster re-epithelialization rate (Wu et al., 2022).

### 2.3 Glycolipids

Glycosphingolipids (GSLs) are the predominant subclass of glycolipids in vertebrates (Schnaar and Kinoshita, 2015). These amphipathic molecules present their complex glycan chains attached to a ceramide (Cer) backbone through a β-glycosidic bond (D'Angelo et al., 2013). Cer can be converted into galactosylceramide (GalCer) within the ER or transported to the cis-Golgi to form glucosylceramide (GlcCer). GalCer can be further processed in the Golgi complex to yield GM4 ganglioside or sulfogalactolipids, also known as sulfatides (Degroote et al., 2004). GlcCer, on the other hand, is converted into lactosylceramide (LacCer) upon reaching the luminal leaflet of the Golgi and TGN membranes. LacCer acts as a metabolic branching point for the formation of over 400 GSLs. Specifically, it can be transformed by B4GALNT1 to produce GA2, by ST3GAL5 to produce GM3, by A4GALT to produce Gb3, and by B3GNT5 to produce Lc3. These products are precursors for the synthesis of GSLs belonging to the asialo, ganglio, globo/iso-globo, and lacto/neo-lacto series, respectively (Russo et al., 2018).

Our understanding of the GSLs present at the ocular surface and their functions is limited. However, some studies have shown that GSLs are integral to various biological processes, including serving as receptors for pathogens and participating in cell migration (Nilsson et al., 2011). Neolactosphingolipids, found in mouse cornea, are involved in epithelial cell migration (Panjwani et al., 1995). Interestingly, the globoside SSEA4, a stemness marker in many tissues (Liang et al., 2010), is identified as a negative marker for Limbal Stem Cells (Truong et al., 2011).

## 3 The Glycocalyx in dry eye disease

Millions of people are affected by dry eye disease (DED) throughout the world, and it remains one of the most frequent causes of visit to an eye clinic. Nonmodifiable risk factors include age, female sex, menopause, and Asian ethnicity, while environmental and behavioral factors such as contact lens wear, electronic device use, air pollution, low humidity, and high altitude further contribute to its prevalence (Stapleton et al., 2017). DED is also associated with ocular conditions like meibomian gland dysfunction and systemic diseases such as Sjögren’s syndrome, diabetes, thyroid disorders, and graft-versus-host disease (Stapleton et al., 2017). TFOS defines DED as “a multifactorial disease of the ocular surface characterized by a loss of homeostasis of the tear film and accompanied by ocular symptoms, in which tear film instability and hyperosmolarity, ocular surface inflammation and damage, and neurosensory abnormalities play etiological roles” (Craig et al., 2017). These factors disrupt tear homeostasis, leading to ocular surface inflammation and alterations in corneal and conjunctival epithelial cells, including changes in their glycocalyx composition (Table 1).

Few studies have explored the role of glycolipids and membrane-bound proteoglycans in dry eye disease. In human tears, levels of two glycolipids, glucosylceramides (GluCers) and monosialotetrahexosylganglioside 3 (GM3), decrease with reduced tear production, though their exact origin remains unclear (Lam et al., 2014). In contrast, numerous studies have focused on mucins, examining changes in gene and protein expression of MAMs in the conjunctiva using impression cytology, as well as alterations in shredded and secreted mucins in tear samples.

### 3.1 Sjögren syndrome dry eye

Sjogren’s syndrome, which causes immune-mediated damage to the lacrimal gland, is primarily associated with aqueous deficient dry eye. Studies on Sjögren Syndrome dry eye have shown conflicting results regarding changes in MAMs. An early study using an antibody against epithelial MAMs in the glycocalyx found decreased levels of these mucins in the bulbar and tarsal conjunctiva of Sjogren syndrome patients, which correlated with increased rose Bengal staining (Pflugfelder et al., 1997). Although the specific transmembrane mucin was not identified, the researchers proposed that the absence of MAMs could lead to the development of squamous metaplasia. A decrease in MUC1 immunoreactivity was later reported in the conjunctival epithelium of patients with Sjögren syndrome dry eye (Jones et al., 1998). Contrary to this, another study detected no changes in MUC1 and MUC4 mRNA levels in bulbar temporal conjunctival impression cytology samples (Argueso et al., 2002). Interestingly, subsequent studies have shown a significant increase in MUC1 and MUC16 mRNA and the shed soluble MUC1 and MUC16 protein in patients with Sjogren syndrome dry eye (Caffery et al., 2010; Caffery et al., 2008). Therefore, the outcomes of studies on changes in MAMs in Sjogren’s syndrome dry eye remain equivocal.

### 3.2 Non-Sjögren dry eye

Like Sjögren syndrome dry eye, studies investigating the changes in membrane-associated mucins in the non-Sjögren dry eye also remain inconclusive. A significant decrease in expressions of MUC1, MUC2, MUC4 and MUC5AC was found in the conjunctival epithelium of patients with non-Sjogren dry eye compared to normal subjects (Corrales et al., 2011). In contrast, other studies have detected no change in MUC1 and MUC16 gene expression in patients with non-Sjogren syndrome dry eye (Caffery et al., 2010; Caffery et al., 2008). A more recent study demonstrated significant upregulation of MUC4 and MUC16 protein expression in the superior and inferior conjunctivas, and MUC1 protein expression in the superior conjunctiva of dry eye patients compared to normal samples (Choi and Tichenor, 2024). Another study found a significant increase in MUC1 and MUC16 mRNA and protein levels in postmenopausal women with non-Sjogren’s mild or moderate dry eye (Gipson et al., 2011). However, a subsequent study could not confirm these findings but rather demonstrated a significant decrease in MUC16 mRNA in postmenopausal women who had significant clinical symptoms of dry eye (Srinivasan et al., 2013).

The alteration of mucin barrier function in dry eye is not solely dependent on the expression of the core mucin proteins but is also significantly influenced by their glycosylation. Using colloid gold-conjugated lectin and TEM, a decrease in sialic acid, N-acetyl-glucosamine, N-acetyl galactosamine, and galactose-N-acetyl-galactosamine was detected in dry eye patients (Versura et al., 1986). Using the H185 antibody directed against the O-acetyl sialic acid epitope of MUC16 (Argueso and Sumiyoshi, 2006), one study found that in normal eyes, this epitope displayed a mosaic immunostaining pattern on apical cells. In contrast, this mosaic pattern was absent in non-Sjögren dry eye patients, replaced by a “starry sky” pattern, with increased binding to goblet cells and a lack of apical cell binding. This “starry sky” pattern correlated with rose Bengal staining (Danjo et al., 1998). Another study, using an antibody directed against the sialylated epitope of MUC1, showed an upregulation of this epitope in patients with mild to moderate dry eye but a downregulation in patients with severe dry eye (Hayashi et al., 2004). Additionally, galectin-3, a glycan-binding protein that binds to N-acetyllactosamine residues, was found to be increased in the tears of dry eye patients (Uchino et al., 2015). It is suggested that alterations in mucin glycosylation lead to a higher presence of this lectin in tears, potentially resulting in adverse effects.

## 4 Contact lens wear and glycocalyx

Only a handful of studies have investigated the effect of contact lens wear on ocular surface glycocalyx. One of the earliest studies examining the effect of soft contact lens wear did not detect any significant differences in gene expression of MUC1, 4, MUC16, or MUC5AC in the conjunctival impression cytology samples or tear protein levels of MUC5AC in long-term tolerant contact lens wearers compared to non-contact lens wearers (Hori et al., 2006). However, another study demonstrated significant alterations in conjunctival mucin gene expression in subjects at 6 months and 12 months after the contact lens wear, and these changes were comparable in subjects wearing either low- or high-water content non-ionic hydrogel contact lenses (Corrales et al., 2009). MUC5AC and MUC16 levels have also been shown to be altered in symptomatic contact lens wearers and correlated with corneal staining (Berry et al., 2008). Wheat germ agglutinin (WGA) is a lectin that binds N-acetyl glucosamine and sialic acid residues in glycosyl side chains. A decrease in fluorescence intensity of fluorescein-conjugated WGA was noted in both symptomatic and asymptomatic contact lens wearers, suggesting that contact lens wear negatively impacts mucin glycan density across the ocular surface (Read et al., 2020). The noted decrease in density was more significant in the lid margin region in symptomatic contact lens wearers. Furthermore, another study found a significant correlation between decreased WGA and tear break-up time (TBUT) in contact lens wearers (Fukui et al., 2016).

Although the use of disposable contact lenses is on the rise, a significant number of people still use reusable lenses. Reusable lenses require multipurpose contact lens solutions for cleaning, disinfecting, and storage purposes. Multipurpose contact lens solutions typically contain a surfactant and a preservative, which potentially could be toxic to the ocular surface epithelial cells. Although no clinical studies have directly compared the effect of contact lens wear on human subjects, *in vitro* studies have tested the potential impact of multipurpose contact lens solutions on mucin expression. Multipurpose contact lens solutions, especially the ones containing boric acid, have been shown to cause a significant decrease in gene expression of MUC1 and a notable reduction in gene expression of MUC4 and MUC16 in cultured human epithelial cells (Imayasu et al., 2010). A similar downregulation in MUC1 and MUC16 was also noted in the corneas of rats exposed to topical application (Tchedre et al., 2013). Some of the solutions also increased the shedding of MUC16 into the culture media, and the exposure of cells to these solutions was associated with an increased infection when exposed to *Pseudomonas aeruginosa* (Gordon et al., 2011).

## 5 The glycocalyx in ocular surface allergies

Common allergic disorders affecting the conjunctiva include seasonal and perennial allergic conjunctivitis, vernal conjunctivitis, and atopic keratoconjunctivitis. Patients with atopic keratoconjunctivitis have decreased levels of MUC5AC, whereas the expression of MUC1, MUC2, MUC4, and MUC16 is increased potentially as a compensation mechanism (Dogru et al., 2008; Hu et al., 2007; Dogru et al., 2006). Similarly, preclinical studies in a mouse model of allergic conjunctivitis also showed a reduced number of goblet cells, with decreased MUC5AC and MUC4 mRNA expression, although the levels showed a rapid recovery (Kunert et al., 2001). In contrast, vernal conjunctivitis patients have an increased number of goblet cells with increased expression of MUC5AC (Aragona et al., 1996). Mass spectrometry analysis of atopic and vernal conjunctivitis patients showed the presence of N-glycan profiles distinct and unique from normal subjects (Messina et al., 2021). A recent study showed an increase in galectin-3 concentrations in tears from patients with vernal conjunctivitis, which might reflect alterations in the glycocalyx composition (Ito et al., 2023).

## 6 The glycocalyx in ocular infection

Ocular surface infections, especially infectious keratitis, can have devastating consequences for vision. The glycocalyx serves as both a physical barrier and a decoy against pathogens. As a barrier, it prevents pathogens from reaching the cell surface. As a decoy, it binds to pathogens to facilitate their clearance. The ocular surface MAMs have been shown to suppress Toll-like receptor signaling and the expression of the proinflammatory cytokines that may modulate the early response to invading pathogens (Menon et al., 2015). The large extracellular domain of MAMs, extending beyond the cell membrane, forms an effective physical barrier against infection. MUC16 and O-glycans have been shown to prevent adherence and infection of corneal epithelial cells by *Staphylococcus aureus* (Ricciuto et al., 2008; Blalock et al., 2007). Some pathogens have adapted to modify the glycocalyx to facilitate infection. For example, strains of *S. pneumoniae,* that can cause ocular surface infection, secrete a metalloproteinase, ZmpC. This enzyme can induce ectodomain shedding of MUC16, resulting in loss of glycocalyx (Menon and Govindarajan, 2013; Govindarajan et al., 2012). Like bacteria, viral infections of the ocular surface can also cause alterations in glycocalyx. The HAdv causes shedding of ectodomain of MUC16 to compromise barrier function (Menon et al., 2016).

MAMs can also bind to pathogens, resulting in their shedding and thus acting as adhesion decoys to facilitate pathogen clearance (Martinez-Carrasco et al., 2021). MUC1 has been shown to bind to various pathogenic bacteria, leading to the shedding of its extracellular domain (Argueso et al., 2021). However, certain pathogens can exploit their ability to bind the glycocalyx to initiate infections. Viruses such as Adenovirus HAdV-D37 and Coxsackievirus A24 variants that cause conjunctivitis have been shown to infect the conjunctiva by binding specific sialic acid components (Mistry et al., 2011; Chandra et al., 2019). In the case of HAdV-D37, the sialylated GD1a ganglioside was identified as a cellular receptor (Nilsson et al., 2011). Thus, sialic acid conjugates have been shown to be effective in preventing HAdV-D37 and Coxsackievirus A24 binding and infection of human corneal epithelial cells (Johansson et al., 2007; Johansson et al., 2020). The proteoglycan SDC1 plays a role in *S. aureus* corneal infection by enhancing bacterial survival at the ocular surface (Hayashida et al., 2011). It also modulates the pathogenesis of herpes simplex virus type-1 (HSV-1) in the cornea by affecting membrane fusion and viral spread (Karasneh et al., 2011). The asialo-GM1 ganglioside acts as a receptor for *P. aeruginosa* in mouse corneas (Hazlett et al., 1993). However, this glycan is absent in rabbit and human corneas (Zhao and Panjwani, 1995).

## 7 The ocular glycocalyx in systemic diseases

Graft-versus-host disease (GVHD) is a common complication of hematopoietic stem cell transplantation, with ocular involvement present in 60%–90% of patients, typically causing severe dry eye. Patients with chronic ocular GVHD also show shorter microvilli with reduced number of membrane-associated proteins (Tatematsu et al., 2012). In a mouse model of bone marrow transplant-induced GVHD, our data showed a significant decrease in the glycocalyx thickness (Shamloo et al., 2019). Specifically, we found a significant decrease in Muc4 and Muc5ac protein levels, as detected by ELISA. These results suggest that alterations in the glycocalyx might be implicated in the pathology of ocular GVHD.

Mucous membrane pemphigoid (MMP) is an autoimmune disorder that causes scarring and primarily targets the mucous membranes of the conjunctiva, nasal cavity, oropharynx, and genitalia. The inflammation and fibrosis typical of MMP result in severe vision loss and even complete blindness (Georgoudis et al., 2019). An increase in polypeptide GalNAc-transferases was noted in patients at the early stage of keratinization due to ocular cicatricial pemphigoid, while the enzyme levels dropped to undetectable levels as the disease progressed to the late stage (Argueso et al., 2003). The noted increase in the early stages of the disease could potentially represent a compensatory response. Additionally, N-glycans are altered in pemphigoid in a manner similar to the changes induced by inflammatory cytokines (Woodward et al., 2019).

Recent findings show that diabetes mellitus also causes damage to the ocular surface, leading to conditions such as dry eye disease and diabetic keratopathy. Data from our lab using mouse models of type 1 and type 2 diabetes mellitus demonstrated a decrease in tear film volume and a concomitant alteration of the glycocalyx, as demonstrated by decreased WGA staining. However, no change in the corneal mucin gene expression was detected in these diabetic mice (Weng et al., 2022) Furthermore, no significant change in rose Bengal exclusion, jacalin staining, and MUC1, MUC4, MUC16 gene or protein expression was noted in stratified human corneal or conjunctival epithelial cells when exposed to high glucose, nor in the gene expression levels of glycosyltransferases (Weng et al., 2023).

## 8 Conclusion

While considerable progress has been made in understanding the ocular glycocalyx, several areas remain unexplored. Historically, the exploration of glycan structures present at the ocular surface has been limited by the availability of suitable techniques. However, recent advancements in mass spectrometry, lectin arrays, and next-generation sequencing are transforming the field of glycobiology. These tools now allow for detailed profiling of glycan motifs and their associated proteins or lipids, uncovering specific glycosylation variations not only across tissue compartments but also within individual cell types (Critcher et al., 2022).

Research on the ocular surface glycocalyx-targeted therapies has primarily focused on mucin production for alleviating dry eye disease. Notably, both diquafosol and rebamipide have been shown to effectively enhance the production of membrane-associated mucins (MAMs) in corneal epithelial cells. Diquafosol increases the gene expression of mucins such as MUC1, MUC4, and MUC16, while also improving tear secretion and corneal barrier function (Uchino, 2018). In contrast, rebamipide boosts mucin production—particularly MUC16—through EGF receptor activation, helping to restore the glycocalyx barrier in dry eye conditions (Uchino et al., 2016). However, studies on the dynamics of MAMs in disease remain inconclusive, and it is not clear whether just enhancing the synthesis of the core protein is enough to restore ocular health.

Notably, less attention has been given to the glycan moieties presented by these mucins and their changes in disease conditions. These moieties, such as poly-lactosamine, are responsible for the functions of lectins like galectin-3 in disease. When these glycan moieties are absent in mucins, galectin-3 is found free in tears, where it interacts with other elements of the glycocalyx, causing epithelial damage. This phenomenon can be exploited for diagnostic purposes (by measuring levels of galectin-3 in tears) or for treatment (by inhibiting galectin-3 with chemical inhibitors). Additionally, there are other glycan moieties, like fucose, that have received little attention. For instance, in a mouse model of dry eye, researchers found aberrant fucosylation that, when inhibited, ameliorated symptoms (Yoon et al., 2021). While it remains unknown whether this occurs in humans, this finding opens the door to targeting terminal glycan modifications for treatment.

Recent advances, such as single-cell RNA sequencing, could help elucidate these potential changes in glycosylation within specific cell types. The glycocalyx of the ocular surface is not limited to mucins, which are only present in the most superficial layer of the epithelia. The underlying cells also present a glycocalyx with a potentially unique composition that has not yet been described. A recent study from one of our labs found that the glycocalyx of limbal progenitor cells, which are responsible for the renewal of corneal epithelium, have particularly low levels of fucose (Woodward et al., 2025). Inhibiting fucosylation in these cells *ex vivo* expanded their replication ability, potentially offering a strategy for treating limbal stem cell deficiency. Additionally, many other cell surface proteins are glycosylated, and the potential alterations of their glycosylation in disease have not been explored. Proteoglycans and glycolipids contribute to cell signaling, structural integrity, and pathogen defense. Yet, their roles in conditions like dry eye disease, ocular allergies, and infections are still poorly defined.

Targeting glycocalyx modifications for therapeutic interventions is a promising area. Emerging tools are enhancing our ability to study glycosylation dynamics and are facilitating the design of glycocalyx-targeted therapies. By utilizing these innovations, we might address longstanding knowledge gaps and uncover new therapeutic avenues to improve patient care in ocular diseases.
